# Acute and chronic viruses mediated by an ectoparasite targeting different developmental stages of honeybee (*Apis mellifera* and *Apis cerana*) brood

**DOI:** 10.3389/fvets.2022.951159

**Published:** 2022-10-06

**Authors:** Zheguang Lin, Nan Zhang, Zhi Wang, Mingliang Zhuang, Qi Wang, Defang Niu, Paul Page, Kang Wang, Qingsheng Niu, Ting Ji

**Affiliations:** ^1^College of Animal Science and Technology, Yangzhou University, Yangzhou, China; ^2^Apiculture Science Institute of Jilin Province, Jilin, China; ^3^College of Forestry, Beihua University, Jilin, China; ^4^College of Food Science and Technology, Jiangsu Agri-animal Husbandry Vocational College, Taizhou, China; ^5^Institute of Agricultural Technology, Suranaree University of Technology, Nakhon Ratchasima, Thailand

**Keywords:** honeybee, deformed wing virus (DWV), Israeli acute paralysis virus (IAPV), *Varroa destructor*, social apoptosis

## Abstract

The health of the western honeybee, *Apis mellifera*, the most crucial pollinator, has been challenged globally over the past decades. An ectoparasitic mite, *Varroa destructor*, together with the viruses it vectored, is generally regarded as the vital pathogenic agent. Although the poor health status of *A. mellifera* compared to its eastern counterpart, *Apis cerana*, has been broadly identified, the underlying mechanism remains poorly understood and comparison between susceptible and resistant hosts will potentially ameliorate this predicament. Here, we investigated the impacts of two widespread viruses—deformed wing virus type A (DWV-A) and Israeli acute paralysis virus (IAPV), mediated by *V. destructor* mite, on the capped developing honeybee brood, in the absence of adult workers, of *A. mellifera* and *A. cerana*, with positive and negative controls. Our results demonstrated that the endogenous viruses imposed limited damage on the hosts even if the brood was wounded. In contrast, the exogenous viruses introduced by ectoparasites triggered variable mortality of the infested brood between host species. Intriguingly, death causes of both honeybee species presented a similar trend: the acute IAPV generally causes morbidity and mortality of late larvae, while the chronic DWV-A typically leads to brood mortality during and after pupation. Notably, the susceptible immature *A. cerana* individuals, supported by higher observed mortality and a lower virus tolerance, serve the interests of the colony and foster the overall survival of a resistant honeybee superorganism. These results improve our understanding of the interactions between viruses carried by ectoparasites and their developing hosts, and the novel insight of weak individuals fostering strong colonies may promote breeding efforts to mitigate the indefensible colony losses globally.

## Introduction

Honeybees, the most important managed pollinating insect, provide vital ecosystem services and contribute to agricultural crop production worldwide (1). However, the health of the western honeybee, *Apis mellifera*, has been challenged globally over the past decades. In contrast, its eastern cousin, *Apis cerana*, thrives in Asia and remains largely unaffected. This distinct health status has been attributed to lower viral loads in *A. cerana* colonies and to their ability to resist an ectoparasitic mite, *Varroa destructor* (2–5). *V. destructor* mites originally parasitized *A. cerana* colonies and did not infest *A. mellifera* until the middle of the last century (6, 7). Since then, an invasive lineage of *V. destructor* rapidly spread worldwide attributed to the global western honeybee trade (7–9).

The ubiquitous ectoparasite *V. destructor*, which punctures the host's integument and feeds on the bee's fat bodies, lives on developing and adult honeybee hosts (10, 11) and grievously injures the honeybee's immune system (12, 13). Without treatment against these mites by beekeepers, *A. mellifera* colonies will usually die within 1–2 years, and as a consequence, most wild and feral *A. mellifera* populations have succumbed to the parasite since it switched hosts nearly a century ago (14, 15). On top of weakening the individuals of a colony, the main damage caused by *V. destructor* is facilitating virus transmission to its hosts, which is regarded as the most severe biotic threat to western honeybees (10, 13, 16). Although the correlation between *V. destructor* and the effects of the viruses it vectors has been intensively studied over the past decade [e.g., (13, 16, 17)], the strategies developed by honeybees to resist and overcome viral diseases remain unclear. Comparing such strategies between susceptible and resistant hosts would therefore allow a better understanding of virus resistance dynamics in honeybee populations.

Although more than 30 honeybee viruses have been identified (18), most of them were of minor importance for *A. mellifera* until the arrival of *V. destructor*, which serves as a vector to many of them, thereby affecting the host's immune system and enabling a drastic change in virus prevalence (13, 16). Among them, two widespread picorna-like viruses, the deformed wing virus (DWV) and the Israeli acute paralysis virus (IAPV), which can be transmitted by *V. destructor*, have triggered much attention worldwide (17, 19, 20). DWV is regarded as a chronic virus with long-term latent infection in the host colonies (21, 22), and IAPV is considered to be an acute virus with extremely virulent pathology, as the affected hosts develop paralysis (23–25). The distinct nature of these two viruses is relevant to investigate their roles, mediated by *V. destructor*, in the infection of susceptible and resistant hosts. The susceptible *A. mellifera* colonies are distributed worldwide, whereas the resistant *A. cerana* colonies can only be found in parts of Asia.

In this study, we took advantage of both host species being sympatric in China to better understand the mechanisms underlying the principal resistance of *A. cerana* to the ectoparasite-associated viruses. The infestation of *V. destructor* on the developing honeybee brood has been reported to cause partial death of both *A. mellifera* and *A. cerana* hosts, i.e., social apoptosis (26, 27). The DWV and IAPV titers of live and dead honeybee broods were quantitatively evaluated. Although three variants of DWV, i.e., DWV-A, DWV-B, and DWV-C, have been described (28, 29), we focused on DWV-A since it occurs most widespread and plays a key role in honeybee health (28, 30). We aimed to examine if the virus proliferation model varied in the two host species that were equipped with different resistance capacities for diseases.

## Materials and methods

### Honeybee colonies

The experimental *A. mellifera* (*n* = 5) and *A. cerana* (*n* = 5) colonies were kept at Wenhui Campus, Yangzhou University, Yangzhou City, southeast China (N 32°23′03″ and E 119°25′35″). The *A. mellifera* colonies were of European origin, without disease-resistant breeding, imported to China a century ago and raised by local beekeepers (31). The *A. cerana* colonies were of indigenous origin (i.e., *Apis cerana cerana*). All the colonies were queenright and kept in Langstroth hives. Two of the *A. mellifera* colonies were not treated against *V. destructor* mites for 6 months and were used as *V. destructor* donor colonies (VDI and VDII).

### Experimental treatment and sample collection

#### *V. destructor* infestation

*V. destructor* mites enter the brood cells of honeybees just before the cells are about to be sealed by workers. Here, we experimentally infested each freshly capped cell with one *V. destructor* mite, previously kept on *A. mellifera* nurse workers that were collected from an apparent healthy colony, for a 2-day dispersal phase (7), following the method established in the laboratory (9). The introduction of the mite into the experimental cell was completed within 6 h after the honeybee larvae were sealed, which has been demonstrated to be consistent with a natural infestation (32). Each batch of *V. destructor* mites was simultaneously used to infest two combs obtained from each honeybee species. We sampled 10 mites in each batch before experimental infestation to assess the viral load of mites. Freshly capped cells of *A. mellifera* and *A. cerana* were infested by the mites derived from VDI and VDII, respectively. For each colony, 15–30 freshly capped cells were used as treatments, and 10–20 control cells were also employed to assess the effect of cell opening. In total, 97 and 93 broods of *A. mellifera* and 109 and 110 broods of *A. cerana* were infested by VDI and VDII, respectively. A total of 65 and 57 broods of *A. mellifera*, and 58 and 55 broods of *A. cerana* were left uninfested.

#### Benign pin-prick

Freshly capped cells were used in this test as well. The wax caps were opened and resealed following the methods of experimental infestation (9). Rather than *V. destructor* introduction, here, we used a sterile capillary needle (Eppendorf TransferTip^®^-R, Hamburg, Germany) to benignly prick the larva body to assess the effect of endogenous viruses on the wounded brood. Four *A. mellifera* and four *A. cerana* colonies, among the five colonies used in the experimental infestation, were employed. For each colony, 15–30 freshly capped cells were used as treatments, and 10–15 control cells were also used. In *A. mellifera*, a total of 57 larvae were pin-pricked and 50 were left as controls. In *A. cerana*, a total of 69 larvae were pin-pricked and 44 were left as controls.

#### Honeybee sample collection

All the treated brood combs were reared in an incubator at 34.5°C and 70% RH (33). One and a half day (1.5 days) later, just before the start of pupation, we collected 5–8 treatment broods and 4–7 control broods with sterile disposable plastic tweezers. The sampling process was done in our laboratory at a room temperature of 30°C, and the sampling time of each group was performed within 30 min, so as to reduce the influence of sampling on the honeybee brood, as well as the state of *V. destructor*. Larvae that appeared black, smelly, or deflated were considered dead larvae. The remaining brood cells were opened and collected 1 day before the expected adult emergence (after 11 days and 10 days for *A. mellifera* and *A. cerana*, respectively). The developmental status of honeybee broods was noted as larva, prepupa, pupa, and pre-emergence adults. The first three stages showing apparent developmental delay were considered as dead brood. Normally developed honeybee brood with dead *V. destructor* mite in the cell was regarded as failed infestation and was not considered in the data analyses. All the sampled honeybee broods were stored at −80°C until RNA isolation.

### Viral load quantification

We extracted the RNA of each honeybee brood and *V. destructor* mite sample using an isolation kit (Tiangen, Beijing, China), following the manufacturer's protocol. Synthesis of cDNA was conducted with RNA products, according to the instructions of the ReverTra Ace qPCR RT Master Mix (Tiangen, Beijing, China). The viral load of each sample was quantitatively evaluated by qPCR using the ChamQ Universal SYBR qPCR Master Mix (Vazyme, Nanjing, China) on the QuantStudio™3 Real-Time PCR System (Applied Biosystems, CA, USA). Three replicates for each sample were performed in a 96-well reaction plate with 2 μL cDNA in a 20 μL reaction volume mixture. For each run, 1 μL forward and 1 μL reverse primers of DWV-A (F: CGTGGTGTAGTAAGCGTCGT; R: TCATCCGTAGAAAGCCGAGT) or IAPV (F: TCGCTGAAGGCATGTATTTC; R: ATTACCACTGCTCCGACACA) were used. The amplification efficiency of the two primers was assessed to be 100.0 and 102.4%, respectively, by gradient dilution of the positive cloned plasmid. Negative (water) and positive (cloned sequence) controls were also included in the reaction. The thermal cycling conditions consisted of 1 min at 95°C followed by 40 cycles of 95°C for 15 s and 60°C for 1 min. A sample was considered virus positive if the melting temperature of the PCR product was similar to the positive controls' and its Ct value was ≤34. Virus titers of positive samples were log-transformed to account for the exponential distribution of the data, and the transformed data were used in the statistical analysis.

### Statistical analyses

Differences in the honeybee brood virus loads among groups were assessed by a non-parametric test followed by a Tamhane *post-hoc* test since the data did not meet normal distribution. The survival rate of honeybee brood between species was analyzed by logistic regression. Student's *t*-test was used to analyze the different virus titers of sampled mites between *V. destructor* donor colonies and other comparisons between the two groups. All the analyses were performed with SPSS Statistics 25.

## Results

### Viral loads in *V. destructor* mites

**Figure 1:**
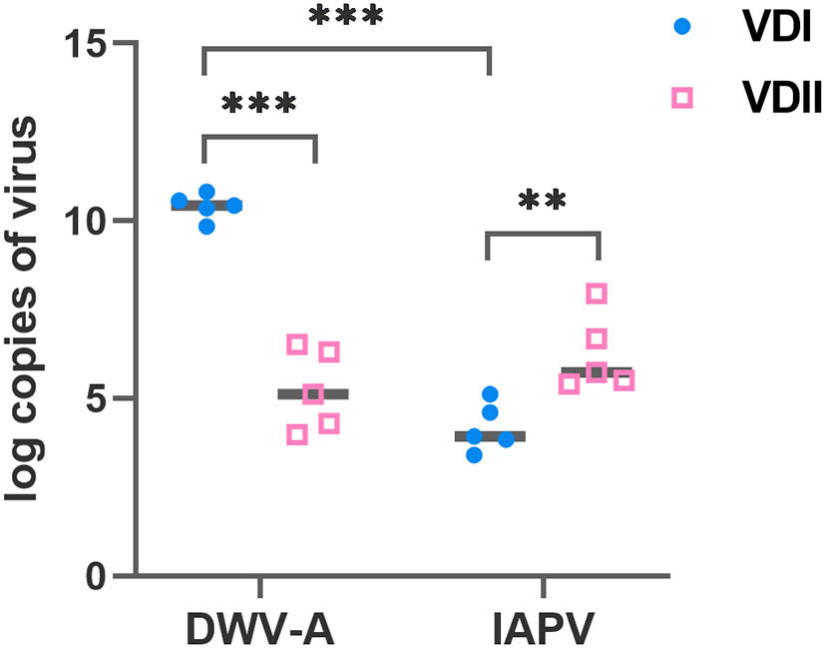
Log-transformed viral loads of *Varroa destructor* mites used for the experimental infestations. Ten mites were collected as a sample before each experiment and five samples were collected. VDI and VDII, two different *V. destructor* donor colonies; DWV-A, deformed wing virus type A; IAPV, Israeli acute paralysis virus; **p < 0.01; ***p < 0.001.

Five batches of *V. destructor* mites, respectively, from VDI and VDII, were sampled before each experimental infestation. The DWV-A load of VDI mites was significantly higher than VDII (Student's *t*-test, *p* < 0.001; Figure 1), and the IAPV load of the former mites was significantly lower than the latter (Student's *t*-test, *p* = 0.007; Figure 1). Meanwhile, for the mites from VDI, DWV-A load was significantly higher than IAPV (Student's *t*-test, *p* < 0.001), while there was no significant difference between the two viral loads in the mites of VDII (Student's *t*-test, *p* = 0.189). Mites from the two donor colonies thus carried different virus baselines before infestation experiments took place.

### Viral loads of honeybee larvae sampled 1.5 days later after capping

**Figure 2:**
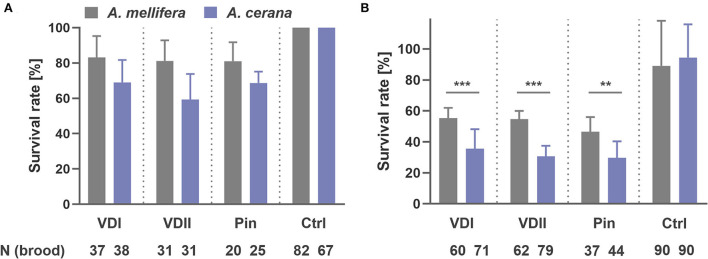
Survival rate of honey bee (*Apis mellifera* and *Apis cerana*) brood sampled at 1.5 days after capping **(A)** and at 1 day before emergence, i.e., 11 and 10 days later after capping, respectively, for *A. mellifera* and *A. cerana* **(B)**. VDI and VDII, brood infested by *V. destructor* mites from two different donor colonies; Pin, the wounded brood pricked by a sterile capillary needle; Ctrl, the pooled control brood with cell opening and resealing operation. **p < 0.01; ***p < 0.001.

A striking difference in brood development was observed. At the first sampling time point-−1.5 days after capping, we collected 37 *A. mellifera* and 38 *A. cerana* larvae infested by VDI with, respectively, 83.8 and 68.4% survival rates (logistic regression, *p* = 0.125; Figure 2A). For VDII, we collected 31 infested larvae of both host species with 80.6 and 58.1% survived brood (logistic regression, *p* = 0.059; Figure 2A). Twenty *A. mellifera* and 25 *A. cerana* pin-pricked larvae were also sampled with 80.0 and 68.0% survival (logistic regression, *p* = 0.369; Figure 2A). More *A. cerana* than *A. mellifera* larvae had died in each treatment type, although the difference between the two species was not significant (Figure 2A). In addition, 31 and 25 for VDI, 27 and 27 for VDII, and 24 and 15 for pin-prick controls of *A. mellifera* and *A. cerana* were, respectively, sampled, and no dead larvae were observed (Figure 2A).

Intriguingly, the viral loads of the two host species displayed a similar trend under the same treatment (Supplementary Figure S1). The infested larvae, both live and dead, did not show a high DWV-A infection (Supplementary Figure S1a). In contrast, IAPV in the infested dead larvae presented significantly higher titers than the other groups (Supplementary Figure S1b), indicating a similar lethal mechanism between the two honeybee species. We thus combined the two sister species with the same treatments in the analyses.

**Figure 3:**
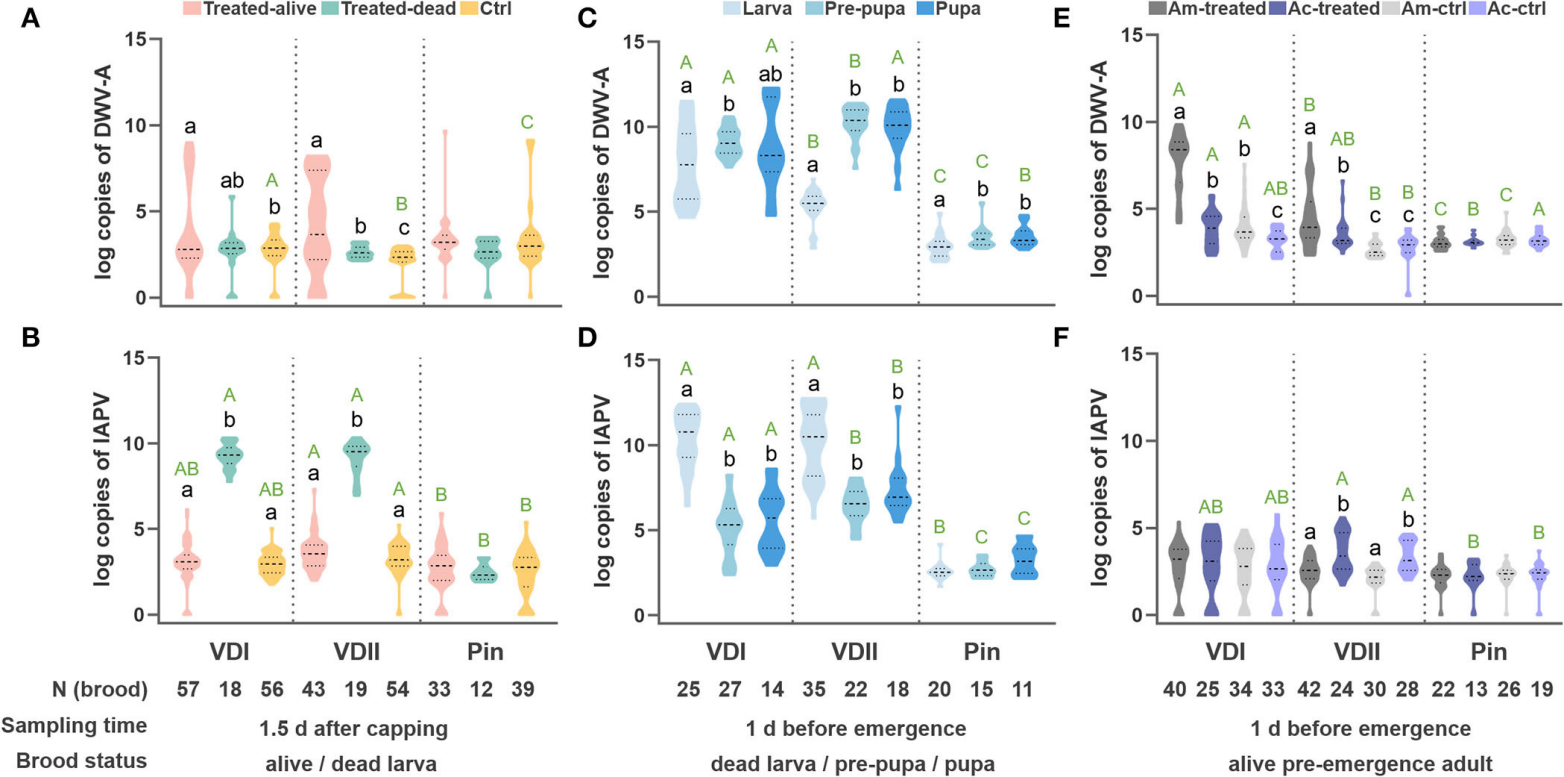
Log-transformed viral loads of the developing honeybee brood. DWV-A load **(A)** and IAPV load **(B)** per honeybee (*A. mellifera* and *A. cerana*) larva sampled 1.5 days after capping. DWV-A load **(C)** and IAPV load **(D)** per dead honeybee brood sampled at 1 day before emergence. DWV-A load **(E)** and IAPV load **(F)** per pre-emergence adult. Different black lowercase letters indicate significant differences within a group (*p* < 0.01) and different green uppercase letters indicate significant differences of DWV-A or IAPV in the samples under the same state (designated as the same color) among VDI, VDII, and Pin groups (*p* < 0.01). VDI and VDII, brood infested by *V. destructor* mites from two different donor colonies; Pin, the wounded brood pricked by a sterile capillary needle; Ctrl, the control brood with cell opening and resealing operation corresponding to each treatment; Am, *A. mellifera*; Ac, *A. cerana*.

The DWV-A loads did not elevate in the dead larvae, while the infested live larvae showed significantly higher DWV-A titers compared to the control (Tamhane, VDI, *p* = 0.022; VDII, *p* < 0.001; Figure 3A). The infested dead larvae displayed significantly higher IAPV loads (Tamhane, VDI, and VDII, *p* < 0.001; Figure 3B), while the infested live ones did not show a higher virus level than the control (Tamhane, VDI, *p* = 0.963; VDII, *p* = 0.155; Figure 3B). Meanwhile, the virus levels of all kinds of brood in the pin-prick group were always low (Supplementary Figure S1; Figures 3A,B), suggesting the limited pathogenicity of endogenous viruses in honeybees.

### Viral loads of honeybee brood sampled 1 day before the expected emergence date

At the second sampling time point, 11 days and 10 days later after capping, respectively, for *A. mellifera* and *A. cerana*, we collected 60 *A. mellifera* and 71 *A. cerana* broods infested by VDI with 66.7 and 35.2% surviving pupae, respectively (logistic regression, *p* < 0.001; Figure 2B). Sixty-two *A. mellifera* and 79 *A. cerana* brood were collected in the VDII infestation group with 67.7 and 30.4% survival (logistic regression, *p* < 0.001; Figure 2B). For the treatment of pin-prick, 37 *A. mellifera* and 44 *A. cerana* brood were available with 59.5 and 29.5% survival (logistic regression, *p* = 0.008; Figure 2B). Significantly more dead brood of the resistant *A. cerana* were found, compared to *A. mellifera*, for all the three treatments (Figure 2B). As for the controls, 89.0 ± 29.2% *A. mellifera* and 94.4 ± 21.5% *A. cerana* brood survived (logistic regression, *p* = 0.568).

The virus loads of the dead brood were first evaluated based on different honeybee species. Again, we found a similar trend between the sister species (Supplementary Figures S1c,d). The dead brood of the two honeybee hosts was thus integrated as above. In this case, DWV-A dominated in the dead pre-pupae and pupae for all three groups, particularly for the brood infested by VDII mites (Tamhane, larva vs. pre-pupa, *p* < 0.001; larva vs. pupa, *p* < 0.001; Figure 3C). Significantly higher DWV-A loads in the dead larvae infested by VDI compared to VDII (Tamhane, *p* < 0.001) may be caused by the higher DWV-A copies in the mites (Figure 1). Moreover, generally significantly higher virus loads of the dead brood of mite-infested than of pin-pricked (Figures 3C,D) resulted from the exogenous viruses transmitted by *V. destructor* as well. Although IAPV load was still high in the dead larva, a significant higher DWV-A level was observed in these samples compared to the dead larvae sampled at 1.5 days (Student's *t*-test, *p* < 0.001 for both VDI and VDII), while this is not the case for pin-pricked samples (Student's *t*-test, *p* = 0.274), indicating the exogenous DWV-A may proliferate by exploiting dead hosts.

Interestingly, the similar trend of virus loads between honeybee species decreased in the pre-emergence adult samples (Figures 3E,F). As the survived larvae were sampled at 1.5 days after capping, the live pre-emergence adults held relatively low viral loads, specifically IAPV, whether it had been treated or not (Figure 3F). IAPV level was rarely high in the dead pre-pupa or pupa (Figures 3B,D,F), suggesting that this virus predominantly targets the early developmental stages before pupation occurs. DWV-A loads in the infested samples were generally higher than in the controls of corresponding species (Tamhane, VDI, *A. mellifera p* < 0.001, *A. cerana p* = 0.026; VDII, *A. mellifera p* < 0.001, *A. cerana p* = 0.015; Figure 3E) and DWV-A loads were superior in the infested *A. mellifera* than in infested *A. cerana* (Tamhane, VDI, *p* < 0.001; VDII, *p* = 0.033; Figure 3E). *A. mellifera* pre-emergence adults infested by VDI mites exhibited a higher DWV-A load than the ones by VDII mites (Tamhane, *p* < 0.001), while the difference was not significant in *A. cerana* individuals (Tamhane, *p* = 0.611). The pricked honeybees did not show higher viral loads than their counterparts (Tamhane, *A. mellifera p* = 0.839, *A. cerana p* = 0.941; Figures 3E,F).

## Discussion

We investigated the effect of two common honeybee viruses—DWV and IAPV, mediated by *V. destructor* mites, on the immature honeybee brood, with positive and negative controls, during the capping period. Consistent with previous findings, the harm of virus infection to the host is limited in absence of *V. destructor* infestation. However, contrary to our expectations, the two viruses direct the morbidity and mortality of immature honeybees at different developmental stages, and the pathogenesis seems accordant between the sister species *A. mellifera* and *A. cerana*. The acute IAPV, vectored by *V. destructor*, typically targets the early stage during capping and may be the principal cause of dead larvae. Mite-carried exogenous chronic DWV normally targets the developing brood since the initiation of pupation and appears to be the potential leading factor of dead pre-pupa and pupa.

Since the viral loads of the pin-pricked samples were always low with no significant difference compared to the controls (Figure 3), the exogenous viruses vectored by the ectoparasitic *V. destructor* mites were considered as the potential culprit for the differences in viral loads observed in our experiments. DWV-A did not massively multiply in any of the 1.5 day samples (Figure 3A), suggesting that DWV-A might not be an acute lethal factor for larvae, which is consistent with the chronic virulent attribute (21, 22). Nonetheless, significantly higher IAPV loads were identified in the infested dead larvae (Figures 3B,D). Two hypotheses were thus proposed for the high IAPV in dead larvae: ① IAPV proliferates after or on the verge of the brood death; ② IAPV leads to the death of the host. If the former holds, the endogenous IAPV would have rapidly multiplied in the pin-pricked samples as well, which does not match the facts. Therefore, acute IAPV is very likely to be a potential causative factor in the rapid death of capped larvae.

Following the above conjecture, the high DWV-A loads detected in pre-pupae and pupae may reflect its fatal role (Figure 3C). The distinctly different virus loads between dead larvae and pre-pupae reveal that DWV-A may exponentially multiply during the brood pupation, while the relatively low IAPV loads in the dead pre-pupae and pupae may reflect the different virus replication modes between acute and chronic viruses. On the other hand, an antagonistic interaction between the two viruses (34, 35) may limit the proliferation of IAPV as a result of high DWV-A infection.

The exogenous viruses vectored by *V. destructor* from two donor colonies led to different viral loads on the infested hosts. In contrast, the viruses of all kinds of brood, including the dead ones, by pin-prick treatment were consistently low. These yields highlight the danger of exogenous viruses mediated by ectoparasitic mites. The corpses of dead larvae, collected 10/11 days after cell capping, had been left in the comb for a few days. Compared to the dead counterparts sampled at 1.5 days, these larva corpses held much higher DWV-A, implying DWV-A may achieve virus proliferation on the dead hosts. However, DWV-A in the pin-pricked dead larvae did not show such differences between the two sampling points. For the dead pre-pupae and pupae, meanwhile, IAPV loads in the infested brood were higher than in the pin-pricked brood (Figure 3D). This divergence between infested and pin-pricked samples may be caused by different virus sources. A finely balanced relationship resides between the endogenous virus and the host (17, 36), while the exogenous viral load may destroy this balance since host immunosuppression induced by *V. destructor* parasitism has been demonstrated to facilitate virus replication (13). The host immunosuppression induced by *V. destructor* parasitism facilitates virus replication as well (12). It hence will be of particular interest in future studies to separate the roles of exogenous virus load and ectoparasite presence to investigate their individual effects on the developing honeybee brood.

In accordance with the dead brood after pupation, the IAPV level is low in the pre-emergence adults (Figure 3F), implying a covert infection in the colonies (23). Covert infection of the chronic and hypovirulent DWV-A is well-known in honeybees (16, 37, 38). Furthermore, the higher DWV-A load in the susceptible *A. mellifera*, compared to the resistant *A. cerana*, suggests a higher tolerance to this virus in the former individuals, which seems to be in contradiction to the weak disease resistance. Indeed, the death of infested and infected brood can limit the proliferation and spread of pathogens to other individuals in the social colony (26, 39). The susceptible immature *A. cerana* individuals, supported by higher observed mortality (Figure 2) and a lower virus tolerance (Figure 3) in our experiments, would thus contribute to the overall survival of a resistant honeybee superorganism, which has been defined as a group immunity of social apoptosis (26, 27). Such findings of achieving social immunity at the expense of individuals are not only instructive for researchers but also could be implemented and integrated into *A. mellifera* breeding programs, which may ultimately alleviate global colony losses and thus contribute to sustain natural and agricultural ecosystems in the long run.

## Data availability statement

The original contributions presented in the study are included in the article/Supplementary material, further inquiries can be directed to the corresponding authors.

## Author contributions

TJ, QN, and ZL conceived the ideas and designed the study. NZ, MZ, and QW carried out the experimental infestations. NZ and ZW performed the pin-prick experiment. ZL, DN, and KW performed the data analyses. ZL, PP, and DN led the writing of the manuscript. All authors contributed critically to the drafts and gave final approval for publication.

## Funding

The financial support was granted by the National Natural Science Foundation of China (32272935 and 31902220 to ZL; 32202743 to DN), the Science and Technology Development Project of Jilin Province (20200402042NC to ZW and QN), the Earmarked Fund for Modern Agro-industry Technology Research System (CARS-44 to TJ, QN, and ZW), the COLOSS Ricola Award for Excellence (ZL), and the Lvyangjinfeng Program of Yangzhou (YZLYJFJH2021YXBS155 to ZL).

## Conflict of interest

The authors declare that the research was conducted in the absence of any commercial or financial relationships that could be construed as a potential conflict of interest.

## Publisher's note

All claims expressed in this article are solely those of the authors and do not necessarily represent those of their affiliated organizations, or those of the publisher, the editors and the reviewers. Any product that may be evaluated in this article, or claim that may be made by its manufacturer, is not guaranteed or endorsed by the publisher.
