# Supplementary material for: Acute and chronic viruses mediated by an ectoparasite targeting different developmental stages of honeybee (Apis mellifera and Apis cerana) brood
Source: Front Vet Sci. 2022 Oct 6;9:951159. doi: 10.3389/fvets.2022.951159 (PMC9583130; doi:10.3389/fvets.2022.951159)
Supplement: Supplementary file 1 [file Data_Sheet_1.pdf]

Supplementary Material

Acute and chronic viruses mediated by an ectoparasite target different developmental stages of honeybee (*Apis mellifera* and *Apis cerana*) brood

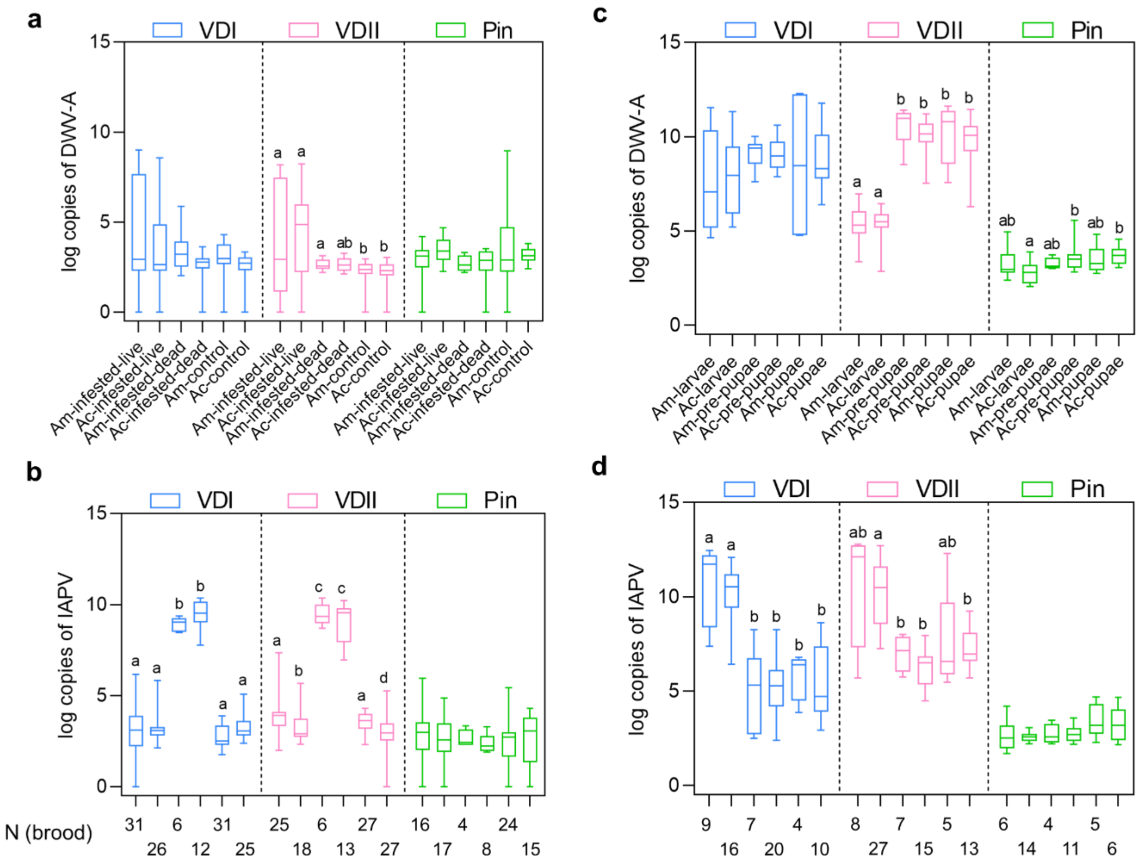

Figure S1. Log transformed viral loads of the developing honeybee (*Apis mellifera* and *Apis cerana*) brood. Deformed wing virus type A (DWV-A) load (**a**) and Israeli acute paralysis virus (IAPV) load (**b**) per honeybee larva sampled at 1.5 d after capping. DWV-A load (**c**) and IAPV load (**d**) per dead honeybee brood sampled at 1 d before emergence. Different letters above the boxes indicate significant differences ($p < 0.05$). Am, *A. mellifera*; Ac, *A. cerana*. VDI and VDII, two different *Varroa destructor* donor colonies.
